# A Novel mRNA-miRNA Regulatory Sub-Network Associated With Prognosis of Metastatic Clear Cell Renal Cell Carcinoma

**DOI:** 10.3389/fonc.2020.593601

**Published:** 2021-01-19

**Authors:** Tianyu Yang, Xiaofen Miao, Zhanxiang Bai, Jian Tu, Shanshan Shen, Hui Niu, Wei Xia, Juan Wang, Yongsheng Zhang

**Affiliations:** ^1^Department of Pathology, The Second Affiliated Hospital of Soochow University, Suzhou, China; ^2^Department of Pathology, Suzhou TCM Hospital Affiliated to Nanjing University of Chinese Medicine, Suzhou, China; ^3^Department of Pathology, The People’s hospital of Hainan Tibetan Autonomous Prefecture, Qinghai, China; ^4^Department of Pathology, Wuzhong People’s Hospital of Suzhou, Suzhou, China

**Keywords:** clear cell renal cell carcinoma, cancer metastasis, bioinformatics analysis, hub genes, miRNAs

## Abstract

**Background:**

Clear cell renal cell carcinoma (ccRCC) is a urinary disease with high incidence. The high incidence of metastasis is the leading cause of death in patients with ccRCC. This study was aimed to identify the gene signatures during the metastasis of ccRCC.

**Methods:**

Two datasets, including one gene expression profile dataset and one microRNA (miRNA) expression profile dataset, were downloaded from Gene Expression Omnibus (GEO) database. The integrated bioinformatics analysis was performed using the (limma) R package, miRWalk, DAVID, STRING, Kaplan-Meier plotter databases. Quantitative real-time polymerase chain reaction (qPCR) was conducted to validate the expression of differentially expressed genes (DEGs) and DE-miRNAs.

**Results:**

In total, 84 DEGs (68 up-regulated and 16 down-regulated) and 41 DE-miRNAs (24 up-regulated and 17 down-regulated) were screened from GSE22541 and GSE37989 datasets, respectively. Furthermore, 11 hub genes and 3 key miRNAs were identified from the PPI network, including FBLN1, THBS2, SCGB1A1, NKX2-1, COL11A1, DCN, LUM, COL1A1, COL6A3, SFTPC, SFTPB, miR-328, miR-502, and miR-504. The qPCR data showed that most of the selected genes and miRNAs were consistent with that in our integrated analysis. A novel mRNA-miRNA network, SFTPB-miR-328-miR-502-miR-504-NKX2-1 was found in metastatic ccRCC after the combination of data from expression, survival analysis, and experiment validation.

**Conclusion:**

In conclusion, key candidate genes and miRNAs were identified and a novel mRNA-miRNA network was constructed in ccRCC metastasis using integrated bioinformatics analysis and qPCR validation, which might be utilized as diagnostic biomarkers and molecular targets of metastatic ccRCC.

## Introduction

Renal cell carcinoma (RCC) is a common cancer worldwide, representing approximately 2–3% of all malignant tumors in adults. It was reported that approximately 63,990 new cases of RCC were diagnosed and more that 14,400 kidney cancer related deaths were found in the United States in 2017 (1, 2). In China, RCC has a rising incidence with about 68,300 new cases of RCC and 25,600 kidney cancer related deaths in 2014 (3). Clear cell RCC (ccRCC) is the most common subtype of RCC and accounts for 80–90% of RCCs. The metastatic dissemination is the most important factor for the prognosis of ccRCC (4). The 5-year survival rate of patients with primary metastasis is about 10%, and that of patients with non-metastasis and advanced metastasis is 70–90% (5). At the time of initial diagnosis, nearly 60% of patients had a local cancer and 20% had distant metastases (4, 6). Since about 30% of patients with ccRCC develop metastatic diseases after surgery, better understanding of metastatic ccRCC is greatly needed. Metastatic spread of ccRCC mainly occurs in bone, lung, liver, or brain. Metastasis of ccRCC is the cause of its high incidence and poor prognosis. More than 80% of patients survived less than 5 years after the diagnosis of distant metastasis (4, 7). Therefore, necessary researches are needed to elucidate the precise molecular mechanisms how metastatic ccRCC occurs and progresses, and establish new molecular-based strategies to better combat this invasive metastatic ccRCC.

With the rapid development of genomics and transcriptome, more and more evidence shows that non-coding RNAs play important roles in the occurrence, development, and metastasis of various cancers. MicroRNAs (miRNAs) are well known for negatively regulating the expression of its target genes *via* transcriptional degradation or repression. Many studies have shown that miRNAs play a key regulatory role in the development of human tumors, and affect the progress of cancer by regulating the expression of genes related to the occurrence and development of the disease (8–10). More and more evidence shows that miRNA-mRNA ceRNA network plays a key role in various cancers including pancreatic cancer, gastric cancer, breast cancer, ovarian cancer, liver cancer, and so on (9–11). However, little is known about the role of miRNA-mRNA ceRNA network in metastatic ccRCC.

In recent years, bioinformatics has become one of the newest fields of biomedical research with the massive amounts of data from high throughout sequencing technologies. Accumulating studies have well documented that bioinformatics analysis have provided a deeper understanding of the aberrant genetic pathways in the development, progression, and metastasis of various human cancers including lung cancer (12), liver cancer (13), ccRCC (14–17), and so on. For example, Chen JY et al. identified 14 potential biomarkers for predicting tumorigenesis and progression of ccRCC using weighted gene co-expression network analysis (14). Zhong MY et al. indicated that tumor necrosis factor-α-induced protein 8 (TNFAIP8) was highly expressed in ccRCC patients and was associated with the development of advanced ccRCC and poor prognosis by database analysis (15). Bioinformatics analysis of the profiling data by Ni D et al. identified FOXO3a as a key factor in ccRCC metastasis (16). Liang B et al. performed bioinformatic analysis and found that E2F1 and E2F2 may serve as valuable diagnostic markers for renal cancer (17). However, systematic analysis of mRNAs and miRNAs in ccRCC is still not enough. In this study, we performed bioinformatics analysis and identified 11 hub genes [fibulin 1(FBLN1), thrombospondin 2 (THBS2), secretoglobin family 1A member 1 (SCGB1A1), NK2 homeobox 1 (NKX2-1), collagen type XI alpha 1 chain (COL11A1), decorin (DCN), lumican (LUM), collagen type I alpha 1 chain (COL1A1), collagen type VI alpha 3 chain (COL6A3), surfactant protein C (SFTPC), and surfactant protein B (SFTPB)] and three key miRNAs (miR-328, miR-502, and miR-504), and established a novel mRNA-miRNA network in metastatic ccRCC. Based on our current findings, it may be used to determine the prognosis of metastatic ccRCC. They may also serve roles in the early diagnosis and therapy of metastatic ccRCC.

## Methods

### Raw Data

The datasets used in our study were downloaded from Gene Expression Omnibus (GEO) (http://www.ncbi.nlm.nih.gov/geo/) of the National Center of Biotechnology Information (NCBI) (18). The included studies were regarding comparing the RNAs in metastatic ccRCC tissues and primary ccRCC tissues. The datasets GSE37989 and GSE22541 were obtained in our study. The dataset GSE37989 included miRNA expression profiles of 9 metastatic ccRCC tissues and 12 primary ccRCC tissues, and the platform used to assess is GPL9081 Agilent-016436 Human miRNA Microarray 1.0 G4472A (miRNA ID version). The dataset GSE22541 included mRNA expression profiles of 24 metastatic ccRCC tissues and 24 primary ccRCC tissues, and the platform for these data is GPL570 [HG-U133_Plus_2] Affymetrix Human Genome U133 Plus 2.0 Array.

### Identification of Candidate Differential Expression Genes and Differential Expression miRNAs

To assess candidate DEGs and DE-miRNAs between metastatic ccRCC and primary ccRCC tissues, R language limma package was performed. The Series Matrix Files of these two datasets were downloaded from GEO database, the data were normalized by the normalizeBetweenArray of limma package, and the analysis of DEGs or DE-miRNAs were performed using the (limma) R package (version 3.6.1).|log_2_FC|>2 and P value <0.05 were set as the cut-off criteria. The volcano plots of DEGs or DE-miRNAs were drawn using (ggplot2) R package (version 3.6.1). The heatmaps of DEGs and DE-miRNAs were drawn by the (heatmap) R package (version 3.6.1).

### Prediction of mRNA–miRNA Interactions

The interactions between DEGs and DE-miRNAs were predicted using miRWalk 3.0 (http://mirwalk.umm.uni-heidelberg.de/) (19, 20), which combined the prediction data of both miRDB (21) and Targetscan (22), and a score of ≧0.95 was considered as the cut-off criterion. Only the target genes included in all of the databases were selected for further analysis.

### GO and KEGG Enrichment Analysis

The significant DEGs were enriched by gene ontology (GO) and Kyoto encyclopedia of genes and genomes (KEGG) using DAVID database (https://david.ncifcrf.gov/) (23). P value <0.05 was considered as statistically significant. The GO items contained three criteria: biological process (BP), cellular component (CC), and molecular function (MF). The bubble plots of pathway enrichment were drawn using the (ggplot2) R package.

### Protein–Protein Interaction Analysis and Hub Genes Identification

The PPI networks between DEGs were constructed using Search Tool for the Retrieval of Interacting Genes (STRING) database (http://string-db.org/) with a combined score ≧0.4 (24, 25). The regulatory network between DEGs and DE-miRNAs was visualized by Cytoscape software (version 3.6.0) (26). Network analysis was carried out to identify the key hub mRNAs with high degrees in the setup network (27, 28), and the top 11 hub genes were visualized in the Cytoscape software based on the node degree.

### Survival Analysis

The Kaplan-Meier plotter database (http://kmplot.com/) (29) was used to evaluate the prognostic values of DEGs and DE-miRNAs. The DEGs and DE-miRNAs were submitted to the database, and the hazard ratio (HR) with 95% confidence interval and logrank p-value were automatically calculated and directly displayed on the webpage. Logrank p-value <0.05 was considered as statistically significant.

### Quantitative Real-Time Polymerase Chain Reaction

Twenty paired ccRCC tissues and the corresponding adjacent non-tumor tissues were obtained from patients with ccRCC. We obtained the written informed consent and the approval from the ethics committee of our Hospital. Total RNA was isolated with the Trizol reagent (Mesgen, China) and the first strand cDNA was synthesized following manufacturer’s protocol. The qPCR reactions were conducted in ABI QuantStudio Dx Real-time PCR Detection Systemusing the SYBR-Green-based method based in MIQE guidelines in our study (30). The human β-actin and human U6 was used as endogenous controls for mRNA and miRNA expression in analysis, respectively. The primers were listed in **Supplementary Table 1**.

## Results

### Identification of Significant Differential Expression Genes and Differential Expression miRNAs in Metastatic ccRCC

**Figure 1:**
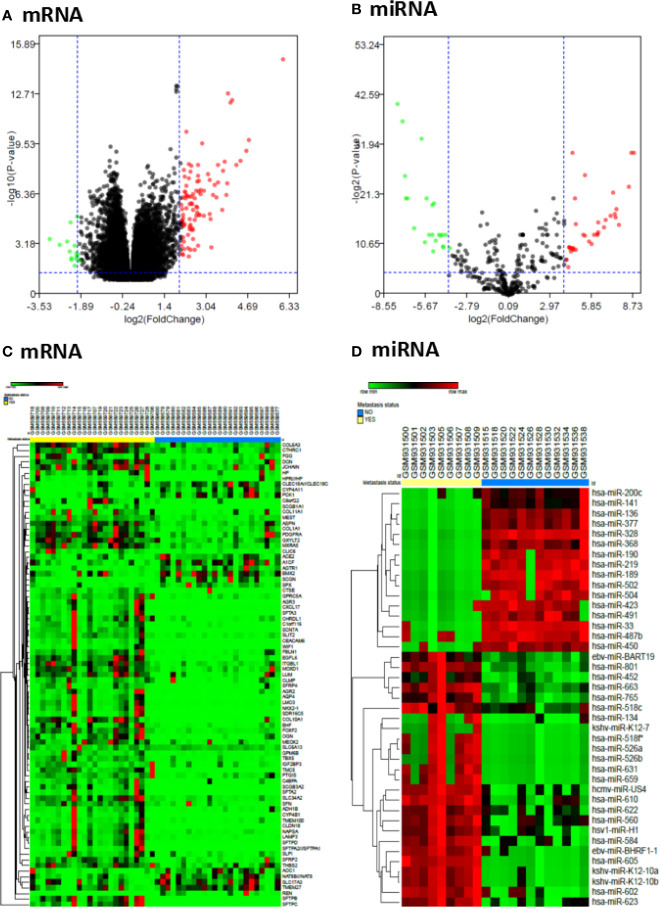
Differentially expressed genes and miRNAs in metastatic ccRCC and primary ccRCC tissues. **(A)** Volcano plot of differentially expressed mRNAs from GSE22541 dataset; **(B)** Volcano plot of differentially expressed miRNAs from GSE37989 dataset; **(C)** Heatmap of differentially expressed mRNAs from GSE22541 dataset; **(D)** Heatmap of differentially expressed miRNAs from GSE37989 dataset.

To search the potential differential expression genes (DEGs) and differential expression miRNAs (DE-miRNAs) in metastatic ccRCC tissues compared with primary ccRCC tissues, we conducted a data mining in Gene Expression Omnibus (GEO) database and two datasets (GSE22541 for mRNAs, GSE37989 for miRNAs) were finally included in our study. Next, the differential expression analysis was performed to find the significant DEGs and DE-miRNAs in each GEO dataset by using the limma package with the criterion of |log2FC|>2 and P value <0.05. Finally, 84 significant DEGs were found in GSE22541 dataset with 68 up-regulated and 16 down-regulated genes, and 41 significant DE-miRNAs were obtained in GSE37989 dataset with 24 up-regulated and 17 down-regulated miRNAs (**Supplementary Tables 2–4**). The volcano plots and heatmaps of significant DEGs and DE-miRNAs were shown in **Figures 1A–D**.

### Functional Enrichment Analysis for the Significant Differential Expression Genes

**Figure 2:**
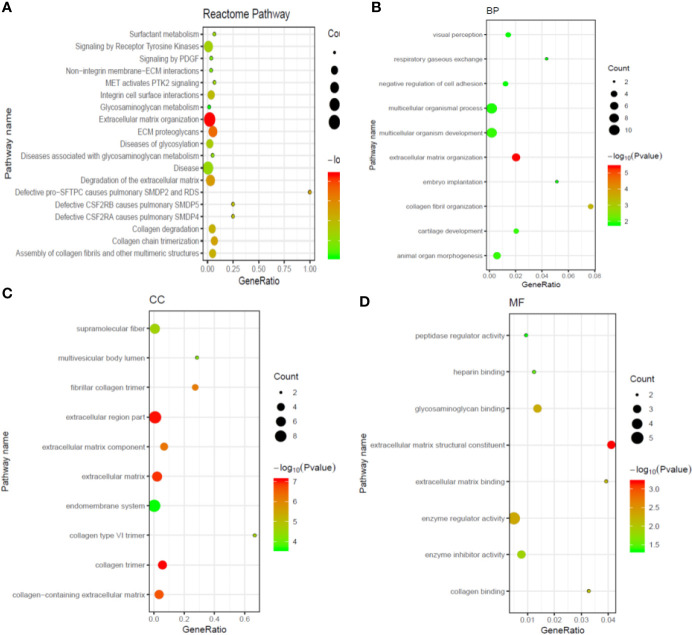
Gene ontology (GO) and Kyoto Encyclopedia of Genes and Genome (KEGG) enrichment analysis for functions of differentially expressed genes in metastatic ccRCC. **(A)** The top 20 most significant KEGG pathway terms; **(B)** The top 10 most significant changes in the GO biological process; **(C)** The top 10 most significant changes in the GO cellular component; **(D)** The top 10 most significant changes in the GO molecular function.

To predict the potential biological functions and related pathways of these DEGs, GO and KEGG assay was conducted. As shown in **Figure 2A**, the DEGs were significant enriched in cancer associated pathways including surfactant metabolism, signaling by receptor tyrosine kinases, signaling by PDGF, non-integrin membrane-ECM interactions, MET activates PTK2 signaling, and so on. Furthermore, the enriched GO functions included visual perception, respiratory gaseous exchange, negative regulation of cell adhesion, multicellular organismal process, extracellular matrix organization in the BP category (**Figure 2B**); supramolecular fiber, multivesicular body lumen, fibrillary collagen trimer, extracellular region part, extracellular matrix component in the CC category (**Figure 2C**); peptidase regulatory activity, heparin binding, glycosaminoglycan binding extracellular matrix structural constituent, extracellular matrix binding in the MF category (**Figure 2D**).

### Protein–Protein Interaction Analysis of miRNA-mRNA Network

**Figure 3:**
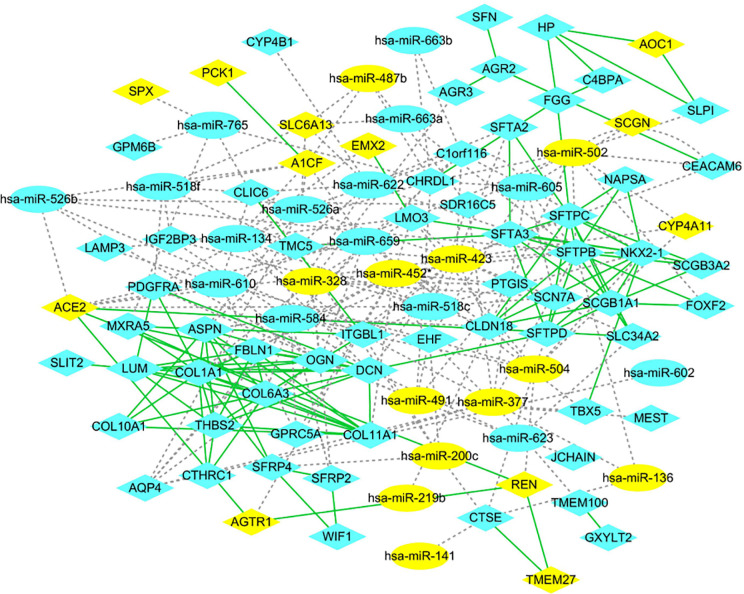
Protein-protein interaction (PPI) network. mRNAs and miRNAs are indicated as ellipsoid and diamond, respectively. The gray dotted line represents the interaction of the miRNA-mRNAs. The green line represents the interaction of the proteins. The yellow indicates high expression, and blue indicates low expression.

**Figure 4:**
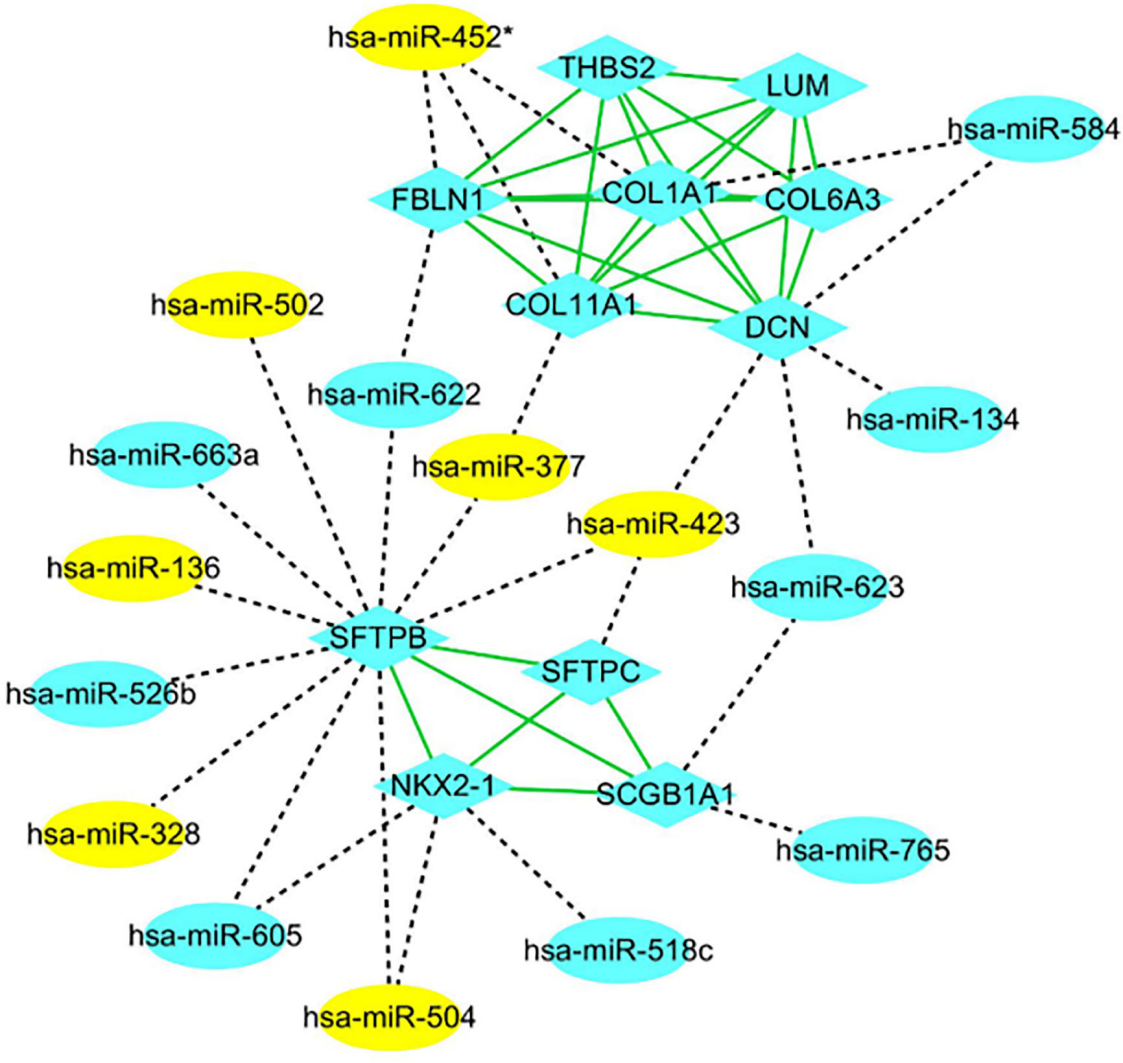
PPI network of hub genes and key miRNAs. mRNAs and miRNAs are indicated as ellipsoid and diamond, respectively. The gray dotted line represents the interaction of the miRNA-mRNAs. The green line represents the interaction of the proteins. The yellow indicates high expression, and blue indicates low expression. * indicates hsa-miR-452-3p.

The PPI networks of the significant DEGs were constructed using STRING database, and the interactions of DEGs and DE-miRNAs were analyzed by miRWalk 3.0 with the criterion of a score of ≧0.95. The network of DEGs and DE-miRNA were re-built and visualized by Cytoscape software. As shown in **Figure 3**, there were 101 nodes in miRNA-mRNA network, which consisted of 84 mRNAs, 27 miRNAs, and formed 283 connections. In addition, the top 11 hub genes were identified from these significant DEGs based on node degree (**Supplementary Table 5**). All the hub genes were up-regulated including FBLN1, THBS2, SCGB1A1, NKX2-1, COL11A1, DCN, LUM, COL1A1, COL6A3, SFTPC, and SFTPB. The regulatory network of hub genes/miRNAs was shown in **Figure 3**. There were 27 nodes in miRNA-mRNA network, which consisted of 11 mRNAs, 16 miRNAs, and formed 53 connections. Among the hub genes/miRNAs network, the down-regulated miRNAs were hsa-miR-452*, hsa-miR-502, hsa-miR-377, hsa-miR-423, hsa-miR-136, hsa-miR-328, and hsa-miR-504, whose target genes were SFTPB, SFTPC, NKX2-1, FBLN1, COL11A1, DCN, and COL1A1, and the up-regulated miRNAs were hsa-miR-518c, hsa-miR-584, hsa-miR-134, hsa-miR-623, hsa-miR-765, hsa-miR-663a, hsa-miR-526b, hsa-miR-622, and hsa-miR-605, whose target genes were COL1A1, DCN, SCGB1A1, NKX2-1, SFTPB, and FBLN1 (**Figure 4**). Based on the classical inverse relationship between miRNAs and target genes, the up-regulated 11 hub genes and their corresponding down-regulated miRNAs were used for further analysis considering that the regulation of genes and miRNAs.

### Validation of Hub Genes and Key miRNAs in Metastatic ccRCC by Prognostic Evaluation

**Figure 5:**
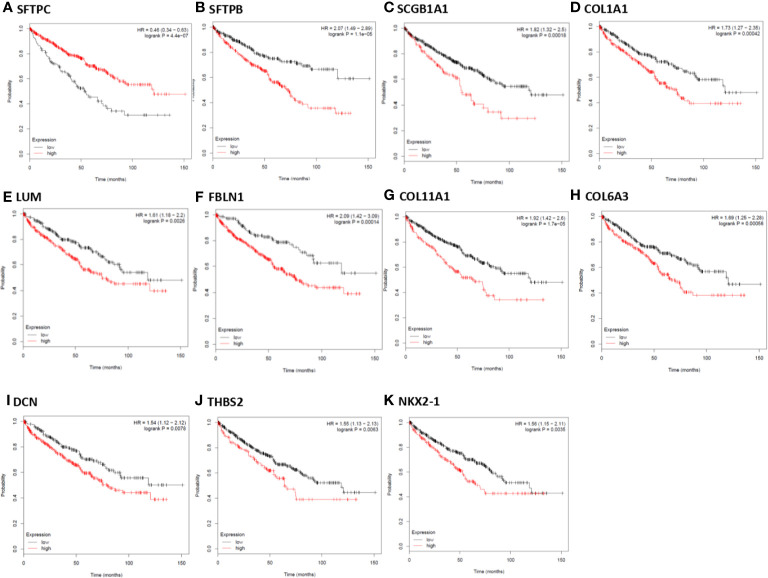
Kaplan-Meier survival analysis for the correlation of 11 hub genes with overall survival of the patients with ccRCC. The vertical coordinate represents the survival probability of ccRCC patients. The red curve represents ccRCC patients with up-regulation of genes, while the black curve represents ccRCC patients with down-regulation of genes.

**Figure 6:**
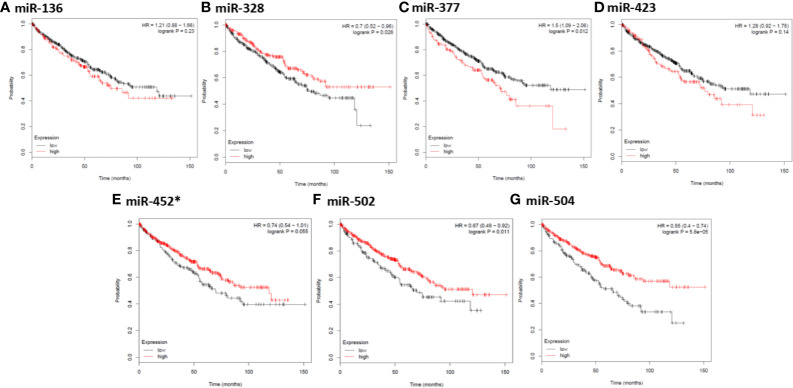
Kaplan-Meier survival analysis for the correlation of seven down-regulated key miRNAs with overall survival of the patients with ccRCC. The vertical coordinate represents the survival probability of ccRCC patients. The red curve represents ccRCC patients with up-regulation of miRNAs, while the black curve represents ccRCC patients with down-regulation of miRNAs. * indicates hsa-miR-452-3p.

In order to further identify key genes in metastatic ccRCC, we detected the prognostic values of the hub genes using Kaplan-Meier plotter database. As depicted in **Figures 5A–K**, the increased expression of ten hub genes (FBLN1, THBS2, SCGB1A1, NKX2-1, COL11A1, DCN, LUM, COL1A1, COL6A3, and SFTPC) indicated poor prognosis in 530 patients with ccRCC (all P < 0.01). Meanwhile, the Kaplan-Meier plotter database was also used to detect the prognostic values of these miRNAs, which were down-regulated in metastatic ccRCC tissues, and could potentially regulate the expression of the above hub genes. As shown in **Figures 6A–G**, the high expression of miR-328, miR-502, and miR-504 exhibited favorable prognostic values in ccRCC with HR values of 0.7 (0.52–0.96, P = 0.026), 0.67 (0.49–0.92, P = 0.011), and 0.55 (0.4–0.74, P = 0.000058), respectively. Combined the above results of expression and survival analysis for key miRNAs, we re-defined the three key miRNAs (miR-328, miR-502, and miR-504) as the key miRNAs.

### qPCR Validation of Hub Genes and Key miRNAs

**Figure 7:**
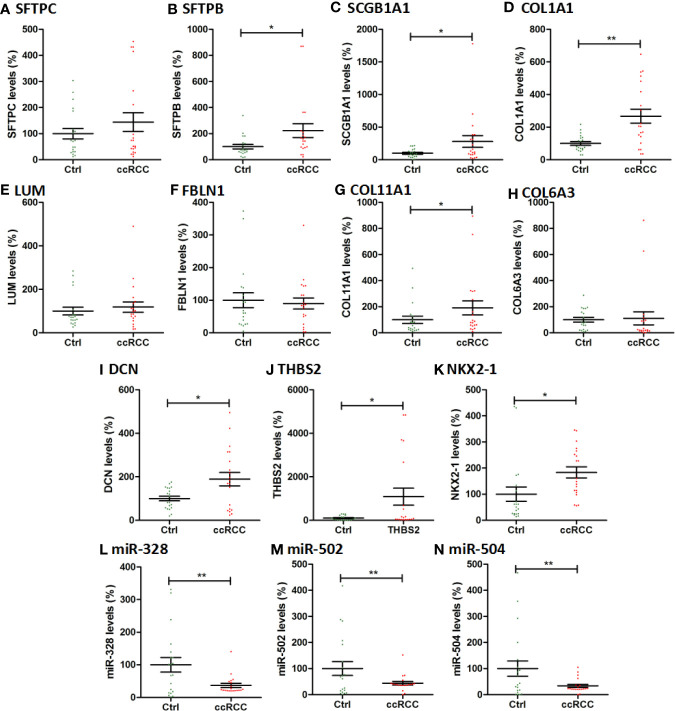
The qPCR results of 11 hub genes and 3 key miRNAs in 20 ccRCC tissues and paired adjacent normal kidney tissues. The vertical coordinate represents the expression levels of genes and miRNAs. Ctrl: Paracancer tissues. * indicates P<0.05; ** indicates P<0.01.

The hub genes and key miRNAs were also validated by qPCR using 20 paired ccRCC tissues and adjacent normal kidney tissues. As shown in **Figures 7A–N**, the expression of SFTPB, THBS2, SCGB1A1, NKX2-1, COL11A1, DCN, and COL1A1 increased significantly in ccRCC tissues compared with adjacent normal kidney tissues (*P *< 0.05, *P *< 0.01) and the expression of SFTPC, FBLN1,LUM, and COL6A3 increased in ccRCC tissues, while the levels of miR-328, miR-502, and miR-504 greatly decreased in ccRCC tissues (*P* < 0.01). Generally, the qPCR results were consistent with that in our integrated analysis.

### Construction of Key mRNA-miRNA Sub-Network in Metastatic ccRCC

**Figure 8:**
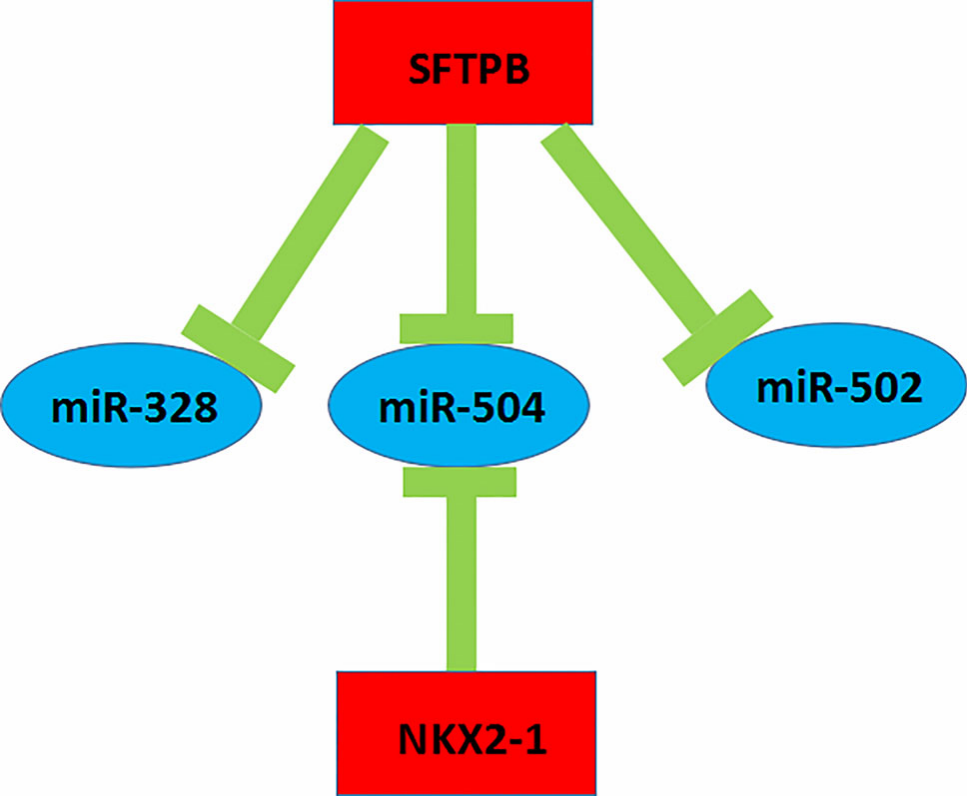
The novel mRNA-miRNA regulatory network associated with prognosis of the patients with ccRCC.

A key mRNA-miRNA competitive endogenous RNA regulatory network in metastatic ccRCC was constructed by a series of data mining and analysis. The network consisted of four mRNA-miRNA pairs (SFTPB-miR-328, SFTPB-miR-502, SFTPB-miR-504, and NKX2-1-miR-504), which was depicted in **Figure 8**. Taken together, we constructed a novel mRNA-miRNA sub-network, SFTPB-miR-328-miR-502-miR-504-NKX2-1, which was obviously associated with the metastatic capacity and prognosis of ccRCC. The sub-network may be developed as promising diagnostic biomarkers or therapeutic targets for metastatic ccRCC in the future.

## Discussion

In recent years, accumulating preclinical and clinical studies have revealed the potential mechanism of metastatic ccRCC, but the incidence and mortality of metastatic ccRCC remain high (16, 31, 32). This is mainly because most studies focus on a single genetic change, or the data based on a single cohort study. In this study, we analyzed two datasets from GEO database and identified 84 DEGs (68 up-regulated and 16 down-regulated), and 41 DE-miRNAs (24 up-regulated and 17 down-regulated). The following GO item and KEGG were used to enrich and analyze differentially expressed genes, and PPI network was constructed. Finally, top 11 hub genes were identified, and a novel mRNA-miRNA regulatory network was constructed, and every RNA in this network exhibited an obvious prognostic value in ccRCC.

After data mining, integrated bioinformatics analysis, and qPCR validation, 11 hub genes were found including FBLN1, THBS2, SCGB1A1, NKX2-1, COL11A1, DCN, LUM, COL1A1, COL6A3, SFTPC, and SFTPB. Among these genes, COL11A1, COL1A1, COL6A3 belong to the collagen family, and are primarily involved in organizing functions through binding to cellular receptors and other components of the extracellular matrix (ECM) (33). COL1A1 is the pro-alpha1 chains of type I collagen whose triple helix comprises two alpha1 chains and one alpha2 chain. COL1A1 is an ECM protein, whose overexpression was linked to breast cancer (34), gastric cancer (34), and colorectal cancers (35). COL1A1 was found to be overexpressed in non-small cell lung cancer (NSCLC) tissues, and COL1A1 correlated with hypoxia markers in NSCLC (36). Moreover, miR-129-5p suppresses gastric cancer cell proliferation, migration, and invasion, by selectively inhibiting COL1A1 (37). COL11A1 is a minor fibrillar collagen. COL11A1 was overexpressed in bowel metastases among patients with ovarian cancer (38), and promoted ovarian cancer progression and is associated with chemo-resistance to cisplatin and paclitaxel in ovarian cancer cells (39). COL6A3 gene silencing inhibits gastric cancer cell proliferation, migration, and invasion while promoting apoptosis through the PI3K-Akt signaling pathway (40). However, the roles of COL11A1, COL1A1, and COL6A3 in the tumorigenesis and metastasis of ccRCC remains to be clarified. Our integrated bioinformatics analysis demonstrated a significant up-regulation of COL11A1, COL1A1, and COL6A3 in metastatic ccRCC tissues, suggesting a proto-oncogene effect of COL11A1, COL1A1, and COL6A3 in ccRCC metastasis. FBLN1 was observed to significantly down-regulated in RCC cell lines and patient tissues through promoter hypermethylation, and FBLN1 over expression led to decreased cell growth, enhanced tumor cell apoptosis, decreased cell motility, and angiogenesis of RCC cells *in vitro* and *in vivo* (41). DCN expression was decreased in RCC tissues compared to adjacent noncancerous tissues and was highly correlated to tumor size (42). In addition, DCN overexpression could inhibit RCC cell proliferation and metastasis by the up-regulating of p21 and E-cadherin (42). Additionally, the roles of THBS2, SCGB1A1, NKX2-1, LUM, SFTPC, and SFTPB in the development and metastasis of ccRCC are unclear. Thus, more researches are needed to further demonstrate the roles and molecular mechanisms of the above hub genes in the metastasis of ccRCC.

A large number of studies have shown that miRNAs play key regulatory roles in the development, metastasis, and progression of human tumors including ccRCC (8–11, 31). MiRNAs not only play the role of proto-oncogenes in tumor cells by promoting the occurrence and development of tumors, but also play a role of anti-cancer *via* inhibiting the oncogenesis and metastasis of cancers. Our integrated bioinformatics analysis identified three down-regulated DE-miRNAs (miR-328, miR-502, and miR-504) in metastatic ccRCC tissues. Moreover, the expression and prognosis of these three miRNAs were further confirmed between ccRCC tissues and normal kidney tissues, suggesting the tumor suppressor role of these miRNAs. Consistent with our current study, recent studies demonstrated the tumor suppressor role of miR-328 and miR-502. miR-328 was found to inhibit the growth of renal cancer cells by regulating cellular adhesion and migration in RCC (43). A recent study showed that knock down of histone methyltransferase SET8, a target gene of miR-502, led to the inhibition of cell proliferation, colony formation, cellular migration, and invasion in RCC cells, implying the anti-cancer activity of miR-502 (44). In addition, miR-504 has been reported to play a tumor suppressor role in the oncogenesis and metastasis of malignant cancers. Liu Q et al. reported that miR-504 suppressed mesenchymal phenotype and invasion of glioblastoma by targeting Frizzled-7 and inhibiting Wnt/β-catenin signaling (45). Ye MF et al. showed that miR-504 was notably down-regulated in non-small cell lung cancer (NSCLC) tissues, and the up-regulation of miR-504 significantly inhibited cell proliferation, cell invasion, and EMT process of NSCLC by directly targeting LOXL2 gene (46). Although multiple studies have suggested the role of miR-504 in cancer development and metastasis, the exact role and mechanisms of miR-504 in ccRCC remains largely unknown yet, which requires further investigation.

Establishment of miRNA-mRNA regulatory networks may provide key evidences to investigate the molecular mechanisms of malignant cancers. For example, Wang WL et al. uncovered key prognostic biomarkers for pancreatic cancer through identification of a novel mRNA-miRNA-lncRNA sub-network (27). Ding BS et al. constructed a potential circRNA-miRNA-mRNA ceRNA network for colorectal cancer (47); Zhang JJ et al. also identified a mRNA-miRNA-lncRNA ceRNA network associated with diagnosis and prognosis of hepatocellular carcinoma (48). The above studies established miRNA-mRNA sub-network utilizing high throughout RNA data in cancer patients. In this study, in order to reveal the possible regulatory network in metastatic ccRCC, we constructed a DE-miRNAs-DEGs regulatory network, in which, SFTPB-miR-328, SFTPB-miR-502, SFTPB-miR-504, and NKX2-1-miR-504 axes may play an important role in the occurrence and metastasis of ccRCC. Intriguingly, most of these key genes have been well investigated in cancers. For example, SFTPB is major component of pulmonary surfactant and is secreted by both alveolar type II and club lung epithelial cells. SFTPB is over expressed in non-small cell lung cancer, especially in lung adeno-carcinoma (49). Moreover, pro-SFTPB in plasma, the precursor of SFTPB, was an independent predictor of lung cancer (50). Clinical studies showed that low NKX2-1 level correlated with unfavorable prognoses in lung adenocarcinoma patients (51). The above studies partially support the accuracy of our bioinformatic analysis. Therefore, more studies are needed to investigate the role of SFTPB, NKX2-1 and their axes in the oncogenesis and metastasis of ccRCC.

In conclusion, our study identified 11 hub genes (FBLN1, THBS2, SCGB1A1, NKX2-1, COL11A1, DCN, LUM, COL1A1, COL6A3, SFTPC, SFTPB) and 3 key miRNAs (miR-328, miR-502, miR-504). Furthermore, we constructed a DE-miRNAs-DEGs regulatory network, which may be helpful for us to clarify the molecular mechanisms of metastasis of ccRCC. Our study provides several key clues to the molecular mechanistic study of metastatic ccRCC.

## Data Availability Statement

The datasets presented in this study can be found in online repositories. The names of the repository/repositories and accession number(s) can be found in the article/**Supplementary Material**.

## Ethics Statement

The studies involving human participants were reviewed and approved by the ethics committee of The Second Affiliated Hospital of Soochow University. The patients/participants provided their written informed consent to participate in this study. Written informed consent was obtained from the individual(s) for the publication of any potentially identifiable images or data included in this article.

## Author Contributions

TY and YZ conceived, designed, and wrote the manuscript. TY, XM, ZB, JT, and JW performed the experiments and analyzed the data. SS and HN helped with the manuscript and data review. All authors contributed to the article and approved the submitted version.

## Funding

This study was supported by funds from Suzhou Science and Technology Development Program (SYS201717), the Second Affiliated Hospital of Soochow University Advance Research Program of the National Natural Science Foundation of China grants (SDFEYGJ1705), and the Advance Research Program of the Second Affiliated Hospital of Soochow University (SDFEYBS1806, SDFEYQN1801).

## Conflict of Interest

The authors declare that the research was conducted in the absence of any commercial or financial relationships that could be construed as a potential conflict of interest.
