# Supplementary material for: A Novel mRNA-miRNA Regulatory Sub-Network Associated With Prognosis of Metastatic Clear Cell Renal Cell Carcinoma
Source: Front Oncol. 2021 Jan 19;10:593601. doi: 10.3389/fonc.2020.593601 (PMC7851075; doi:10.3389/fonc.2020.593601)
Supplement: Supplementary file 1 [file DataSheet_1.pdf]

Supplementary Table 1 The primers for qRT-PCR.

Name	Sequence, 5'-3'
RT primers	
U6	CGAGCACAGAATCGCTTCACGAATTTGCGTGTCAT
miR-328	GTCGTATCCAGTGCAGGGTCCGAGGTATTCGCACTGGATACGAC ACGGAA
miR-502	GTCGTATCCAGTGCAGGGTCCGAGGTATTCGCACTGGATACGAC TGAATC
miR-504	GTCGTATCCAGTGCAGGGTCCGAGGTATTCGCACTGGATACGAC GATAGA
qRT-PCR primers	
U6	F: CGAGCACAGAATCGCTTCA;<br>R: CTCGCTTCGGCAGCACATAT
miR-328	F: GTCGTATCCAGTGCAGGG;<br>R: CTAATCTGGCCCTCTCTGCC
miR-502	F:GTCGTATCCAGTGCAGGG;<br>R: CGACTAATGCACCTGGGCAA
miR-504	F: GTCGTATCCAGTGCAGGG;<br>R: CGACTAGACCCTGGTCTGCA
actin	F: CACCATTGGCAATGAGCGGTTCC;<br>R: GTAGTTTCGTGGATGCCACAGG
SFTPB	F: TGGAGCAAGCATTGCAGTG;<br>R: ACTCTTGGCATAGGTCATCGG
FBLN1	F: AGAGCTGCGAGTACAGCCT;<br>R: CGACATCCAAATCTCCGGTCT
THBS2	F: CGTGGACAATGACCTTGTTG;<br>R: GCCATCGTTGTCATCATCAG
SCGB1A1	F: TTCAGCGTGTCATCGAAACCC;<br>R: ACAGTGAGCTTTGGGCTATTTTT
NKX2-1	F: ATGTACCGGGACGACTTGGAA;<br>R: CAATGCCTGTCAGGGCTAGAA
COL11A1	F: TGGTGATCAGAATCAGAAGTTCG;<br>R: AGGAGAGTTGAGAATTGGGAATC
DCN	F: ATGAAGGCCACTATCATCCTCC;<br>R: GTCGCGGTCATCAGGAACTT
LUM	F: TAACTGCCCTGAAAGCTACCC;<br>R: GGAGGCACCATTGGTACACTT
COL1A1	F: GAGGGCCAAGACGAAGACATC ;<br>R: CAGATCACGTCATCGCACAAC
COL6A3	F: AAGCCTGTGTATCGTGGAG;<br>R: TTAAGGCATTGGTCCCAAC

SFTPC	F: CACCTGAAACGCCTTCTTATCG;<br>R: TTTCTGGCTCATGTGGAGACC

F: forward primer; R: reverse primer

Supplementary Table 2 The significant dys-regulated genes and miRNAs in the GSE22541 and GSE37989 datasets.

Items	Up/down	mRNAs or miRNAs
mRNAs	up	CTSE ADH1B AGR2 AGR3 AQP4 ASPN C1orf116 C4BPA C8orf22 CEACAM6 CHRDL1 CLDN18 CLIC6 CLMP COL11A1 COL1A1 COL6A3 CTHRC1 CXCL17 CYP4B1 DCN GFL6 EHF FBLN1 FGG FOXF2 GPM6B GPRC5A GXYLT2 HP HPR IGF2BP3 ITGBL1 JCHAIN AMP3 LMO3 LUM MEOX2 MEST MOXD1 MXRA5 NAPSA NKX2-1 OGN PDGFRA TGIS SCGB1A1 SCGB3A2 SCN7A SDR16C5 SFN SFRP2 SFRP4 SFTA2 SFTA3 SFTPA1 SFTPA2 SFTPB SFTPC SFTPD SLC34A2 SLIT2 SLPI TBX5 THBS2 TMC5 TMEM100 WIF1
mRNAs	down	REN AOC1 PCK1 SPX CYP4A11 SCGN SLC17A3 NAT8 A1CF SLC6A13 EMX2 NAT8B CLEC18A ACE2 TMEM27 AGTR1
miRNAs	up	kshv-miR-K12-7 hsa-miR-134 hsa-miR-801 hsa-miR-765 ebv-miR-BART19 hsa-miR-526b hsa-miR-663 hsa-miR-560 hsa-miR-622 hsa-miR-526a hsa-miR-518f* hsa-miR-623 hsa-miR-602 hsa-miR-584 hsa-miR-631 ebv-miR-BHRF1-1 hsa-miR-610 hsa-miR-659 hcmv-miR-US4 hsv1-miR-H1 hsa-miR-518c*
miRNAs	down	kshv-miR-K12-10b kshv-miR-K12-10a hsa-miR-605 hsa-miR-33 hsa-miR-136 hsa-miR-377 hsa-miR-141 hsa-miR-190 hsa-miR-219 hsa-miR-450 hsa-miR-328 hsa-miR-368 hsa-miR-189 hsa-miR-200c hsa-miR-487b hsa-miR-491 hsa-miR-502 hsa-miR-504 hsa-miR-452* hsa-miR-423

Supplementary Table 3 The top 10 significant up-regulated and down-regulated genes in the GSE22541 dataset.

Gene symbol	Up/Down	logFC	adj.P.Val	P.Value	t	B
AGR3	Up	3.65	2.04E-05	1.27E-08	6.84	9.601316
SCGB3A2	Up	3.72	7.75E-06	2.98E-09	7.25	10.95381
SFTPA1	Up	3.79	1.62E-04	4.09E-07	5.86	6.346604
AQP4	Up	3.81	5.36E-05	7.45E-08	6.34	7.942409
SCN7A	Up	3.86	9.33E-10	1.37E-13	1.02E+01	20.17364
TMC5	Up	3.94	2.90E-09	5.30E-13	9.76	18.93829
EGFL6	Up	4.01	2.21E-09	3.64E-13	9.88	19.28136
SFTPC	Up	4.67	6.04E-07	1.33E-10	8.14	13.85068
SFTPB	Up	6.01	2.60E-11	9.51E-16	1.17E+01	24.64632
SFTPA2	Up	6.33	9.33E-10	1.24E-13	1.02E+01	20.2587
REN	Down	-3.53	1.79E-04	5.13E-07	-5.79	6.13459
AOC1	Down	-3.2	8.09E-03	2.61E-04	-3.94	0.323056
PCK1	Down	-2.78	1.44E-02	6.61E-04	-3.64	-0.53232
SPX	Down	-2.5	1.05E-02	3.83E-04	-3.82	-0.03053
CYP4A11	Down	-2.4	2.50E-02	1.67E-03	-3.33	-1.38077
SCGN	Down	-2.35	1.92E-03	2.31E-05	-4.69	2.575254
SLC17A3	Down	-2.32	4.36E-02	4.26E-03	-3	-2.22496
NAT8	Down	-2.32	5.31E-02	5.91E-03	-2.88	-2.51703
A1CF	Down	-2.2	1.48E-02	6.88E-04	-3.63	-0.56907
SLC6A13	Down	-2.19	8.60E-02	1.32E-02	-2.57	-3.22755

Supplementary Table 4 The top 10 significant up-regulated and down-regulated miRNAes in the GSE37989 dataset.

miRNA_ID	Up/Down	logFC	adj.P.Val	P.Value	t	B
hsa-miR-631	Up	7.09665	7.93E-06	3.71E-07	7.3204	6.4023
ebv-miR-BHRF1-1	Up	7.24064	2.57E-07	6.02E-09	9.4602	10.5885
hsa-miR-610	Up	7.35661	6.33E-06	2.85E-07	7.449	6.6712
hsa-miR-659	Up	7.42476	9.68E-06	4.70E-07	7.2065	6.1623
hcmv-miR-US4	Up	7.50016	2.83E-06	1.07E-07	7.9339	7.6652
hsv1-miR-H1	Up	7.67754	3.25E-05	2.17E-06	6.4894	4.612
hsa-miR-518c*	Up	8.34274	1.05E-07	2.26E-09	10.0174	11.5807
kshv-miR-K12-10b	Up	8.54007	6.75E-10	9.71E-12	13.5622	17.0759
kshv-miR-K12-10a	Up	8.68186	6.75E-10	7.72E-12	13.7299	17.3052
hsa-miR-605	Up	8.7323	9.41E-17	3.18E-19	32.6129	33.3473
hsa-miR-33	Down	-8.54896	2.72E-08	5.39E-10	-10.8746	13.0335
hsa-miR-136	Down	-7.70284	4.59E-13	2.48E-15	-20.8464	25.192
hsa-miR-377	Down	-7.38992	6.29E-12	4.53E-14	-17.9731	22.3863
hsa-miR-141	Down	-7.17411	2.16E-08	3.88E-10	-11.078	13.3658
hsa-miR-190	Down	-7.12729	6.17E-07	1.58E-08	-8.9307	9.6092
hsa-miR-219	Down	-7.03493	6.17E-07	1.74E-08	-8.8787	9.5111
hsa-miR-450	Down	-6.54667	4.97E-05	3.58E-06	-6.2603	4.1029
hsa-miR-328	Down	-6.44905	9.41E-17	3.38E-19	-32.5156	33.2956
hsa-miR-368	Down	-6.02449	8.30E-11	7.47E-13	-15.5382	19.6298
hsa-miR-189	Down	-5.71146	6.17E-07	1.95E-08	-8.8181	9.3963

Supplementary Table 5 Topology parameters of top 11 hub genes (degree ≧4) in the miRNA-mRNA regulatory network

Gene	Closeness	Betweenness	Degree
FBLN1	0.44827586	0.09323077	8
THBS2	0.37142857	0	6
SCGB1A1	0.45614035	0.12492308	5
NKX2-1	0.41269841	0.09076923	6
COL11A1	0.44827586	0.09323077	8
DCN	0.49056604	0.28235897	10
LUM	0.37142857	0	6
COL1A1	0.38235294	0.02287179	8
COL6A3	0.37142857	0	6
SFTPC	0.44827586	0.01723077	4
SFTPB	0.56521739	0.54923077	13
